# Impact of Pre-Transplantation Psychological Counseling in Improving the Mental Well-Being of Patients on Hemodialysis

**DOI:** 10.3389/fpsyt.2021.594670

**Published:** 2021-02-09

**Authors:** Qianyun Zhao, Sen Zhang, Ran Yu

**Affiliations:** ^1^Blood Purification Center, The Second Hospital of Anhui Medical University, Hefei, China; ^2^Department of Nephrology, The Second Hospital of Anhui Medical University, Hefei, China

**Keywords:** kidney transplantation, hemodialysis, negative emotions, interventions, end-stage renal disease

## Abstract

**Background:** Patients who are on hemodialysis (HD) and are waiting for kidney transplantation encounter various psychological issues.

**Objective:** The current research aimed to compare the effectiveness of regular nursing care with that of nursing care coupled with dedicated psychological counseling in patients who were on HD before they underwent kidney transplantation.

**Methods:** Baseline data were collected 1 month before kidney transplantation in patients of both the control (patients who received general nursing care between August 2011 and June 2015) and intervention (patients who received nursing care and psychological counseling between June 2015 and July 2018) groups. The Mental Status Scale in Non-Psychiatric Settings (MSSNS) was administered to assess and record the psychological status. Clinicodemographic and end-stage renal disease (ESRD)-related details, including duration of dialysis, causes for ESRD, the number of dialysis sessions performed before transplantation, and MSSNS scores, were recorded and compared between the groups.

**Results:** A total of 37 patients were enrolled, including 19 in the control group and 18 in the intervention group. The number of dialysis sessions performed before transplantation was 143 (26, 564) and 227.5 (39, 767), and dialysis duration was 20.4 ± 14.5 and 14.4 ± 12.1 months in the intervention and control groups, respectively. There was no significant difference in baseline negative emotions between the two groups (*P* > 0.05). The psychological intervention group reported significantly lower anxiety, depression, anger, and loneliness scores than the control group (*P* < 0.05).

**Conclusion:** Psychological counseling before kidney transplantation in patients on HD could reduce their negative emotions and enhance mental well-being.

## Introduction

Kidney transplantation is a primary therapeutic option for patients with end-stage renal disease (ESRD). Patients who are on hemodialysis (HD) and are waiting for kidney transplantation encounter various psychological issues, which often get unnoticed or under-recognized by the treating clinicians and the family of patients. Anxiety and depression are known to be prevalent among patients with ESRD, particularly among females and older patients (1).

Hitschfeld et al. reported that lung transplant recipients with a low score in the “Psychosocial Assessment of Candidates for Transplant Scale” could reflect weak or borderline psychosocial candidacy and develop a higher likelihood of mortality (2). Such findings may be extrapolated to other types of solid organ transplants as well. Especially for patients with ESRD requiring transplantation, the familial, social, economic, and marital statuses were reported to affect their mental and physical well-being besides the influence of disease adaptation (3). In Chinese renal transplant recipients, depression seems to be a widespread phenomenon. Employment, financial pressure, residential locality, and social support affect their mental well-being more profoundly. Lin et al. suggested that Chinese renal transplant recipients should have a routine evaluation of depression during follow-up visits. Individualized care by collecting information on the depressive symptoms and other related parameters may ease the psychological issues in these patients and help improve their quality of life (QoL) (4).

For patients with ESRD, especially for those undergoing living related donor kidney transplantation, the primary causes of psychological irregularities include long-term HD, suffering from the disease, heavy economic burden, and the very concern about surgery (5). The negative emotions can result in disorders of the vegetative nervous system and endocrine functions, adversely affecting the immune function of patients, as well as the safety of surgery. Mental status, such as depression, may also affect physical well-being. Hence, placing more patients on transplant waiting lists is considered to reduce depression and provide a higher QoL with a better outcome during dialysis therapy (6). Early diagnosis of depression and the implementation of appropriate treatment may enhance the QoL in waiting list patients (7). Interestingly, Alavi et al. demonstrated that the prevalence of depression and anxiety was higher among patients on HD than among patients who received renal transplantation. The choice of treatment is a significant contributor to the variance in the activities of daily living and depression scores among ESRD patients (8).

Kidney transplantation is a critical life-saving incident, but it also constitutes reflective psychological, relational, and social changes for the patient, donor, and their families. Self-orientation, knowledge on the transplantation surgery, and support from family and society are critical factors to influence the surgery outcomes, which can eventually alter the psychological stability of the patient. Therefore, an adequate pre-transplant psychosocial assessment provides a deeper understanding of the patients' psychological health status and helps to develop a suitable customized post-transplant psychotherapy plan for coping strategies (9).

There is a significant gap in the evidence available on the impact of psychological interventions in patients with ESRD. Most of the psychological interventions would not include psychological consoling (10, 11). The current research aimed to compare the effectiveness of regular nursing care with that of nursing coupled with dedicated psychological counseling in patients with ESRD before they underwent kidney transplantation.

## Materials and Methods

### Patients

Patients from the Second Hospital of Anhui Medical University, Hefei, Anhui Province, China who were on HD and received routine nursing care (between August 2011 and June 2015; control group) and nursing care coupled with dedicated psychological counseling (between June 2015 and July 2018; interventional group) before undergoing kidney transplantation were recruited by a non-randomized controlled intervention design. The inclusion criteria were: (1) ≥18 years old, (2) clinically confirmed ESRD, (3) required allogeneic kidney transplantation, (4) patients who are actively receiving HD, (5) otherwise stable vital signs and physical conditions to tolerate transplantation surgery, and (6) clear consciousness. The exclusion criteria were: (1) severe infections and other pre-surgery complications; (2) history of mental disorders, neuropathy, and traumatic brain injury; (3) hearing and language disorders; (4) history of drug and alcohol dependence; and (5) severe heart, brain, lung, and other organ damages. The study protocol was thoroughly reviewed and approved by the Second Hospital of Anhui Medical University Ethics Committee, and written informed consent was obtained from all patients before enrollment.

### Interventions

The control (2011–2015) group received routine nursing, regular dialysis (no. of sessions depending on individual pathological condition), dietary guidance, and routine clinical care. The control group did not do any intervention treatment and only did a questionnaire survey after the follow-up. On the other hand, the intervention (2015–2018) group received dedicated psychological counseling in addition to the treatments that the control group received.

Approximately a month before kidney transplantation, the “Mental Status Scale in Non-Psychiatric Settings” (MSSNS) was administered to patients of both the groups to assess and record the psychological status.

### The Mental Status Scale in Non-psychiatric Settings

This scale contained four key dimensions: anxiety (13 items), depression (10 items), anger (eight items), and loneliness (seven items), which are rated by a 5-point Likert scale (0–4 points). The total score of 38–152 points had different ranges for these dimensions: 13–52 points for anxiety, 10–40 points for depression, 8–32 points for anger, and 7–28 points for loneliness. A higher score indicated significant negative emotions. The Cronbach coefficient α of the scale was 0.933, which had good reliability and validity.

### Psychological Counseling

Trained nurses (two nurses supervised by a head nurse) formed the psychological counseling team. Based on the scores obtained from the MSSNS scale, the psychological problems of the patients in the intervention group were analyzed to understand the causes. Group discussions were arranged to develop weekly psychological counseling programs, and each lasted 20–30 min during the HD treatment.

To be specific, the intervention depended on the personalized customization of patients. Patients with psychological problems were screened out according to the MSSNS scale and scored which part (anxiety, depression, anger, and loneliness) goes higher. Then, the nurses pointed out the reason that caused the negative mood of the patients by communicating with them and adopted corresponding nursing on the basis of that. For example, if the patient felt lonely, during the dialysis treatment, more companionship and communication were provided by the nurses. Moreover, the nurses also reminded the family members to pay attention to the patient's mood swings and give more care and companionship.

### Data Collection and Outcomes

Clinicodemographic and ESRD-related details, such as gender, age, marital status, educational level, occupation, duration of dialysis, causes for ESRD, number of dialysis sessions performed before transplantation, and baseline MSSNS scores, were recorded 1 month before transplantation for all patients, and the scored dimensions of the MSSNS were compared between the groups. After 1 month of treatment (till the day before surgery), all patients were recorded the MSSNS scores again.

### Statistical Analysis

SPSS 20.0 software (IBM, Armonk, NY, USA) was used for statistical analyses. Continuous data were tested with the Kolmogorov–Smirnov test for normal distribution. Normally distributed continuous data were expressed as mean ± standard deviation (SD), and non-normally distributed continuous data were expressed as median (range). The counts were expressed as percentage and compared using the chi-square test. The measurement data were expressed as mean ± SD. For comparison within groups before and after treatment, paired samples *t*-test was used, whereas the comparison between the groups was performed by independent samples *t*-test. *P* < 0.05 was considered statistically significant.

## Results

### Sociodemographic Characteristics

A total of 37 patients were enrolled, including 19 in the control group and 18 in the intervention group. The patients were mostly men (46.7 and 53.3%), and the mean ages were 35.3 ± 6.7 and 40.4 ± 12.0 years, in both the groups, respectively. The sociodemographic features of the two groups were comparable (*P* > 0.05) (Table 1).

**Table 1:** Sociodemographic characteristics.

**Item**	**Intervention group (*n* = 18)**	**Control group (*n* = 19)**	***P***
Male, n (%)	14 (77.8)	16 (84.2)	0.693
Age (year), mean ± SD	35.3 ± 6.7	40.4 ± 12.0	0.123
Marital status, *n* (%)			0.66
Married	16 (88.9)	15 (78.9)	
Unmarried	2 (11.1)	4 (21.1)	
Educational level, *n* (%)			0.714
College level and above	13 (72.2)	11 (57.9)	
High school/technical secondary school level	2 (11.1)	4 (21.1)	
Junior high school level and below	3 (16.7)	4 (21.1)	
Medical insurance, *n* (%)	18 (100.0)	18 (94.7)	xx
Occupation, *n* (%)			<0.001
Yes	1 (5.6)	4 (21.1)	
No	17 (94.4)	15 (78.9)	

### Clinical Characteristics

The primary causes of ESRD for kidney transplantation did not differ significantly between the groups. The median number of dialysis sessions performed before transplantation was 143 (26, 564) and 227.5 (39, 767), and dialysis duration was 20.4 ± 14.5 and 14.4 ± 12.1 months in the control and intervention groups, respectively (Table 2).

**Table 2:** Clinical characteristics.

**Item**	**Intervention group (*n* = 18)**	**Control group (*n* = 19)**	***P***
Causes for ESRD, *n* (%)			>0.999
Primary glomerulonephritis	16 (88.9)	17 (89.5)	
Anaphylactoid purpura	0	1 (5.3)	
Nephrotic syndrome	2 (11.1)	1 (5.3)	
Dialysis duration (months), mean ± SD	20.4 ± 14.5	14.4 ± 12.1	0.175
Number of dialysis (times), median (range)	227.5 (39, 767)	143 (26, 564)	0.175

### MSSNS Score

There were no significant differences in the control and intervention groups on the absolute values of total MSSNS score (*P* = 0.530), anxiety (*P* = 0.089), depression (*P* = 0.841), anger (*P* = 0.389), and loneliness scores (*P* = 0.344) in baseline. After intervention, the absolute values of total MSSNS score, anxiety, depression, anger, and loneliness scores in the intervention group were statistically lower than those in the control group (all *P* < 0.001). The absolute scores difference between before and post-intervention in the intervention group was greater than that in the control group (*P* < 0.001) as shown in Table 3. Anxiety was common in this group, mainly because kidney transplantation was poorly understood, and patients worried about the outcome of transplantation. Five patients in this group had enormous economic pressure and worried about treatment costs. One patient had heart enlargement and hypertrophy due to long-term dialysis, and two reported hypotension during dialysis. All of these patients developed depression. After intervention, the patient's heart was significantly smaller, cardiac function improved, and the patient met the transplantation criterion. The patient's depression gradually improved. Three patients showed anger by breaking some objects or verbal venting, which might be due to their impatience and long-term suffering from dialysis. After intervention, the patients' anger is significantly reduced. Some other patients on HD felt lonely and seemed isolated from their relatives.

**Table 3:** The absolute values for each item of MSSNS and the differences from the baseline before transplantation.

**Item**	**Intervention group (*n* = 18)**	**Control group (*n* = 19)**	***P***
**Anxiety**			
Baseline	27.6 ± 2.1	28.7 ± 1.9	0.089
Post-intervention	21.3 ± 3.1	25.7 ± 2.3	<0.001
Decline from baseline	6.3 ± 2.3	3.0 ± 1.9	<0.001
**Depression**			
Baseline	21.3 ± 1.2	21.2 ± 2.2	0.841
Post-intervention	15.6 ± 2.3	19.0 ± 2.3	<0.001
Decline from baseline	5.7 ± 1.8	2.2 ± 1.7	<0.001
**Anger**			
Baseline	15.7 ± 1.3	16.1 ± 1.7	0.389
Before transplantation	10.8 ± 1.0	13.7 ± 2.8	<0.001
Decline from baseline	4.8 ± 1.6	2.4 ± 1.7	<0.001
**Loneliness**			
Baseline	16.2 ± 1.9	16.7 ± 1.7	0.344
Post-intervention	13.7 ± 2.5	16.2 ± 2.3	<0.001
Decline from baseline	2.4 ± 1.5	0.5 ± 1.1	<0.001
**Total score**			
Baseline	80.6 ± 3.9	81.5 ± 4.9	0.530
Post-intervention	61.4 ± 5.1	74.5 ± 7.2	<0.001
Decline from baseline	19.1 ± 3.6	7.0 ± 5.1	<0.001

*All data were shown as mean ± SD*.

## Discussion

The present study involved the comparison of nursing alone and nursing with psychological counseling of patients who were on HD before kidney transplantation. The results suggested that well-planned psychological counseling of patients would ease the negative emotions in this patient population and result in mental well-being.

In recent years, with the advances in medical technology, kidney transplantation has become the treatment of choice of patients with ESRD. The incidence of uremia in China is gradually increasing, and the number of patients undergoing living, related, donor kidney transplantation is on the rise. Although kidney transplantation can effectively prolong the life of patients, the trauma and drug therapy associated with the surgery may induce tolerability issues in few patients. The cost of kidney transplantation is expensive in most settings, and the other crucial factors, such as postoperative adverse reactions, complications, and possible rejection due to immunosuppression, may adversely affect the physical and psychological health of patients (12). Especially for patients undergoing living related donor kidney transplantation, more significant psychological pressure is usually present during the perioperative period. The donors often have fear, tension, hesitation, and ambivalence, and the recipients often have anxiety, fear, sensitivity, and guilt (13). Different degrees of anxiety and depression will be present after surgery for patients undergoing living related donor kidney transplantation, and such negative emotions are negatively correlated with the QoL of patients (14).

Since June 2015, this department had performed psychological interventions to patients who were on HD before kidney transplantation. The overall psychological states for the treatment group after the intervention, including anxiety, depression, anger, and loneliness scores, were significantly lower than those in the control group, indicating that comprehensive psychological interventions could improve the mental well-being and ease out the psychological issues of patients before kidney transplantation. Patients undergoing kidney transplantation lack subjective initiative in self-management. When rejection or complications occur at a later point in time or when donors have physical and mental reactions, the patients develop distinct negative emotions of anxiety, tension, guilt, and self-accusation. Therefore, preoperative psychological interventions should be conducted to mobilize their initiative for self-management.

Fu and Coyte reported in a large cohort retrospective study of data from 5,922 kidney transplant recipients from deceased donors that predialysis psychosocial conditions were associated with the increased relative risk of post-transplant death (15). Patients with vs. without a history of non-adherence, which might be due to pre-transplant mental health concerns, tended to have a higher but statistically nonsignificant cumulative incidence of acute rejection (23.3 vs. 13.6%) and death-censored graft failure (16).

Hence, education and counseling on mental health should be provided to patients timely before surgery, and professional nurses should help patients to establish an excellent psychological defense mechanism and change negative emotions into positive psychological experiences. Previous studies have also revealed that correct and positive nursing interventions can significantly improve the quality of nursing and eliminate the patient's negative psychological state. In this study, the preoperative dialysis plans were developed per the patient-specific conditions, including hypotension, coronary heart disease, and heart failure, which could significantly improve the success rate of kidney transplantation. Adequate dialysis has always been the most basic and essential aspect of ESRD treatment, which could prolong the survival time. It can provide a good preparatory state and a stable health condition before surgery, which would ultimately improve the QoL.

In general, the nursing diagnoses and interventions seem limited in the care of kidney transplant recipients. A holistic approach to treatment and management offers physical, psychological, and social well-being of the patients (17). In some settings, the involvement of a multidisciplinary health care approach is recommended for the good psychological and emotional health of the transplant recipient. The continuous support during the post-transplant adaptation process seems highly crucial for the mental well-being of the patient (18). Scheel et al. utilized the “Transplant Effects Questionnaire” to evaluate the coping process after renal transplantation. Their results suggested a conditional structure with a weak and negative association of mental QoL with emotions, such as worries, guilt, and responsibility, but positively with disclosure. However, this psychological processing does not affect treatment adherence (19).

In order to improve mental health, it is imperative to evaluate the depression and stress-coping types at a very early stage, maybe in the dialysis period itself. A well-developed educational intervention supported by need-based counseling would benefit these patients (20). HD patients were reported to have fewer functional coping strategies and had more mental health issues than those who underwent renal transplantation (21). However, the number of patients who receive psychopharmacologic therapy seems very less. Martinelli et al. reported that only 22% of patients received such treatment and received psychological and psychiatric support (22). The present study may pose some limitations, mainly its sample size. Despite that, the results obtained in the current work may add significant value to the existing literature on the importance of pre-transplant psychological counseling in patients with ESRD.

There are some limitations to this study. This is a single-center study with a small sample. Furthermore, the patients in this study were enrolled in a non-random way, which would result in the bias to interfere with the evaluation of effectiveness. Therefore, a large sample size and multi-center randomized controlled trial is needed in the future.

In conclusion, the psychological intervention in patients with ESRD before kidney transplantation could have a positive impact on mental well-being and favorable outcomes postoperatively by improving their negative emotions.

## Data Availability Statement

The original contributions presented in the study are included in the article/supplementary material, further inquiries can be directed to the corresponding author/s.

## Ethics Statement

The study protocol was thoroughly reviewed and approved by The Second Hospital of Anhui Medical University Ethics Committee. The patients/participants provided their written informed consent to participate in this study.

## Author Contributions

QZ designed and implemented this research. SZ was responsible for the writing and revision of the paper. RY participated in the data collection and processing. All authors contributed to the article and approved the submitted version.

## Conflict of Interest

The authors declare that the research was conducted in the absence of any commercial or financial relationships that could be construed as a potential conflict of interest.
